# Irish Dancing Injuries and Associated Risk Factors: A Systematic Review

**DOI:** 10.3390/ijerph20126190

**Published:** 2023-06-20

**Authors:** Ana Rita Póvoa, Cláudia Maria Costa, Sérgio Simões, Ana Morais Azevedo, Raul Oliveira

**Affiliations:** 1 Egas Moniz School of Health and Science, 2829-511 Almada, Portugal; 2 Hospital Garcia de Orta, 2805-267 Almada, Portugal; claudia.maria.costa@hgo.min-saude.pt; 3 CiiEM—Centro de Investigação Interdisciplinar Egas Moniz, 2829-511 Caparica, Portugal; 4 La Trobe Sport and Exercise Medicine Research Centre, La Trobe University, Melbourne 3086, Australia; 5 The Australian Ballet, Melbourne 3006, Australia; 6 Interdisciplinary Centre for the Study of Human Performance, Neuromuscular Research Lab, Human Kinetics Faculty, University of Lisbon, 1499-002 Cruz Quebrada-Dafundo, Portugal; roliveira@fmh.ulisboa.pt

**Keywords:** Irish dance, injuries, risk factors, injury prevention, epidemiology, prevalence, incidence, surveillance system

## Abstract

Irish dance is growing in popularity, evolving to a more athletic and demanding dance style. The aim of this study is to conduct a systematic review, previously registered with PROSPERO, to identify the prevalence, incidence, and the injury pattern among Irish dancers and analyse the associated risk factors. Six online databases and two dance-specific science publications were searched systematically. Studies were included if the patterns of injuries among Irish dancers were evaluated or the factors associated with injury were analysed, published in English or Portuguese, in peer-reviewed scientific journals. Four reviewers assessed the quality and level of evidence using the Downs and Black criteria and a modified Oxford Centre of Evidence-Based Medicine 2009 model, respectively. Eleven articles were included, eight of Level 3c (cross-sectional) and three of Level 3b (prospective). Mean DB percentage score was 63% ± 7.2%. Prevalence ranged from 72.2% to 92.6%, affecting mostly the foot/ankle complex. Only two articles reported incidence, which ranged from 3.4 to 10.6 injuries/1000 h danced depending on injury definition. Psychological factors, elite level, and insufficient/poor sleep were associated with musculoskeletal injury. Injury prevalence and incidence is high in Irish dancers, with the foot and ankle being more affected. Due to heterogeneity in injury definitions, methods, and populations, along with the need for improvement in studies quality, recommendations were made for future research.

## 1. Introduction

Irish dance has gained popularity all over the world and has become an extremely athletic and competitive activity [1,2]. This dance style is characterised by ballistic and explosive movements, numerous jumps, and rapid leg movement while the upper body remains upright, with the arms held at the side [3,4]. 

Irish and ballet dance styles have similar technique characteristics such as an ex-tended landing posture at initial contact (e.g., hip and knee extension, foot and ankle plantar flexion and erected spine); however, ballet dancers have to smoothly “roll through their feet” upon landing using the “demi-plié” (hip and knee flexion and ankle dorsiflexion) [5,6]. In contrast, Irish dancers land on a plantar flexed foot position with an extended knee, not taking advantage of the “plié” [1,7], which has been associated to dissipate landing forces [6] as well as to pliable landing [8].

Due to the demanding characteristics of Irish dance and increasing competitiveness, the risk of injury might increase, particularly of the lower extremities. However, there is only one systematic review (published in 2013) regarding injuries in Irish dancers that included two studies with limited quality and level of evidence [9]. Since then, there has been an increasing amount of research regarding the epidemiology of Irish dance injuries [3,4,10,11,12,13,14,15,16].

A new systematic review including the most recent research will allow to further advance knowledge in this growing dance style, which is crucial for the development of effective injury prevention and intervention programs [17,18]. 

The main purpose of this study was to conduct a systematic review to identify the injury pattern among Irish dancers and analyse the associated factors, comprising the most recent available evidence.

## 2. Materials and Methods

### 2.1. Protocol and Registration

This systematic review was conducted following the recommendations of the PRISMA Statement for Reporting Systematic Reviews and Meta-Analysis [19]. The protocol was registered in advance with the PROSPERO International prospective register of systematic reviews (registration number CRD42020163302).

### 2.2. Eligibility Criteria

Observational, prospective, retrospective, and cross-sectional studies published in English and Portuguese in peer-reviewed scientific journals were included. Studies were included if they evaluated the pattern of injuries among Irish dancers and/or analysed factors associated with injury among Irish dancers. Case reports of injuries among Irish dancers or focusing on a specific injury or body part were excluded.

The main outcome measures included injury frequency (prevalence, incidence, injury rates); occurrence pattern of musculoskeletal injuries (which injuries, its nature, severity, and anatomic distribution); mechanism of injury; main associated factors. To include all relevant information, no restrictions were applied to types of outcome measures nor to definitions of injury or type of data collection tools.

### 2.3. Search Strategy

Studies were identified by searching the following selected electronic databases: SCOPUS, PubMed, Web of science, Scielo, Lilacs and B-on from their year of inception up to February 2020. The search strategy was also extended to two dance-specific science publications (Journal of Dance Medicine and Science and Medical Problems of Performing Artists). Additionally, a manual search of the references of articles identified in the database searches were screened. The last search was run in September 2020. One reviewer (ARP) developed and conducted the search. The following search keywords were used: “irish” AND “danc*” AND “injur*” OR “pain”. To search all databases, search terms were altered when necessary, e.g., “dance” or “dancer” or “dancing” instead of “danc*”. In the search strategy for PubMed, the following query was used: irish danc* AND (injur* OR pain).

### 2.4. Selection Process

Screening was performed by reviewer ARP. Citations were imported to Mendeley Reference Manager (version 1.19.4) and duplicates were removed. When titles and abstracts revealed potential for inclusion, full-text articles were obtained and independently assessed for eligibility by four reviewers (ARP, SS, AMA and RO), all physiotherapists with experience in systematic reviews. Disagreements between reviewers were resolved by consensus.

### 2.5. Data Collection Process

A data extraction form was created (Appendix A) and pilot-tested on each included paper by one reviewer (ARP). Corrections were discussed with another reviewer (RO) to improve data collection. Data were independently extracted and cross-checked by three reviewers (ARP, AMA and RO) and recorded in an Excel spreadsheet. Disagreements between individual judgements were resolved by consensus between reviewers.

For this systematic review, prevalence was defined as the proportion of existing cases and period prevalence was defined as the proportion of dancers that reported the condition of interest at any time during that given window. Incidence was defined as the number of new injuries in the population that develop during a defined period [20]. In articles where prevalence or period prevalence was not provided, this was calculated via the proportion of injured dancers during the study time frame.

### 2.6. Study Quality Assessment

There is a lack of a single tool for assessing susceptibility to bias of observational epidemiological studies [21]. Existing tools for assessing risk of reporting biases in studies have many limitations; for example, they are not developed to appraise different study designs, i.e., each tool can be applied to a limited types of study designs. There is also no one single tool that appraises all sources of bias [22]. Risk of bias and quality of included studies were assessed using the Downs and Black (DB) quality assessment tool [23] and were conducted by four independent reviewers (ARP, CMC, SS and AMA). With the purpose to reduce checklist limitations in assessing the risk of bias [24], besides the DB checklist, the reviewers were also asked to access any potential source of bias in the included studies.

The checklist was modified due to the design of the included studies. Twelve items were not applied to the included studies (items 4, 8, 9, 13–15, 17, 19, 23–24, 26–27) as they are related to intervention studies, and items 5, 21, and 22 were omitted for studies that did not provide an independent control group. The maximum achievable score for the included studies was 16. Scores were then presented as percentage scores [25].

The level of evidence of each study was categorised based on the Differential Diagnosis/Symptom Prevalence Study guidelines by the Oxford Centre for Evidence-Based Medicine (OCEBM) 2009 model [26]. The 2009 model was considered more appropriate for the current dance science literature regarding the hierarchy of evidence and it has been applied in previous systematic reviews [27,28]. As defined in the inclusion criteria, Levels 1a, 2a, 3a (systematic reviews), 4 (case series) and 5 (opinion-based papers) were not included. Cross-sectional studies were included in Level 3, since this was the study design more frequently identified; thus, the 2009 OCEBM model was modified adding Level 3c [27,28]. To access selective reporting bias, every article was examined for missing outcomes listed in the Section 2 that were not presented in the results [19].

## 3. Results

**Figure 1:**
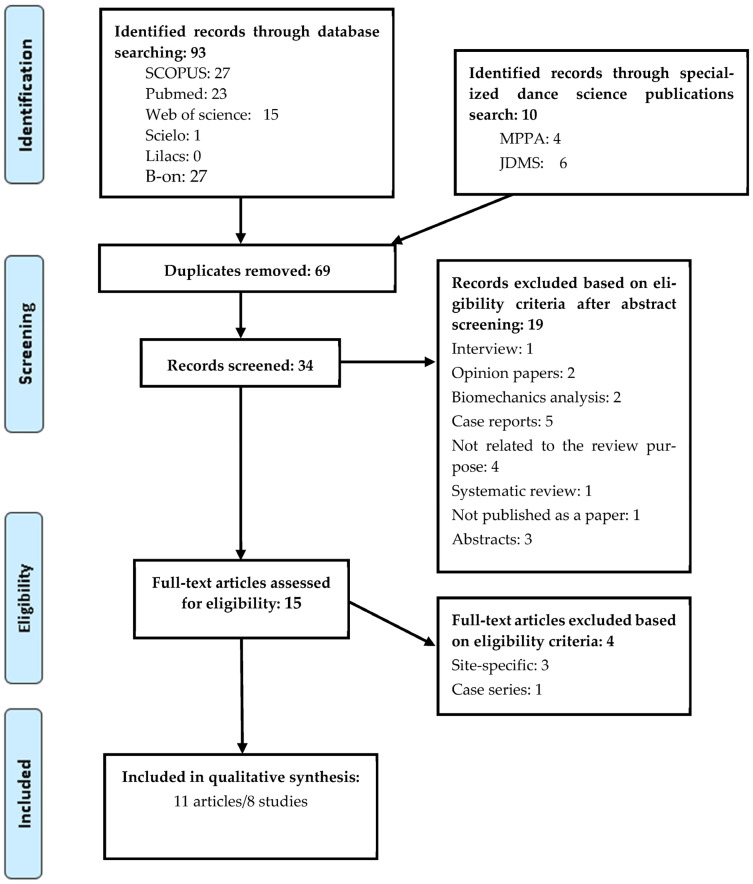
PRISMA Flow Diagram of Study Selection.

Results of the selection process are displayed in Figure 1. After full-text screening, based on eligibility criteria, four articles were excluded, three were focused on a specific body area [7,29,30] and one was a case series study [31]. The manual search of the references of articles identified in the initial searches yielded no further articles. As findings of three studies were reported in six publications, a total of eleven articles were considered eligible for review, representing eight studies (for more information, see Table S1).

### 3.1. Study Characteristics

Characteristics of the 11 included articles are detailed in Table S1. Study design, data source and participant details are summarized in Table 1. Four articles did not present an explicit injury definition [2,10,12,15], four articles used injury definitions based on time-loss from dance, rehearsal, performance, training, or competition [3,13,14,32]. One article used a medical attention injury definition [16] and two articles defined injury as “any pain or injury that impacted upon their ability to dance” [4,11]. 

The included studies involved 865 participants and the mean number of participants per study was 108 (range of 21–255). The overall sex ratio was 5.7/1 (female/male), and the total number of females reported was 735 (range of 20–247) and that of males was 130 (range of 0–67). Most participants were recruited from Irish dance schools [3,10,11,14,15], followed by University-level institutes of dance [4,12], performing productions, the An Coimisiun le Rinci Gaelacha and social media [3,14] or during a competition [32]. Medical records were obtained from an academic sports medicine centre of a single company [2] and clinics at the investigators’ hospital [16].

### 3.2. Risk of Bias and Level of Evidence

Table 2 presents the level of evidence and DB scores after adjustments for consensus between reviewers. The mean DB percentage score and standard deviation was 63% ± 7.2%. Four articles did not report or provide any injury definition [2,10,12,15], and none of included articles described confounders in each group of subjects to be compared (where applicable). Also, two articles did not provide measures of dispersion [2,16], and two did not report probability values [2,15].

Included articles failed in external validity as they had non-random sampling methods: convenience sampling [4,11,12,32] and volunteer sampling [3,10,13,14,15]. Furthermore, small sample size might be a limitation in the included studies. Only one study mentioned a sample size calculation to determine an adequate sample size, but the way in which the calculation was performed was not described [15]. 

Most articles accomplished internal validity concerning study bias, although one did not describe statistical analysis [2]. Plus, in two articles it was not possible to determine whether the main outcome measures used were valid and reliable, as not all the used questionnaires were validated [14,32] and one had a recall period of the entire career [14].

None of included articles met the DB criterion for reporting adjustment for confounding. Three studies used multivariate regression modelling in statistical analysis [3,11,13]; however, it is not clear whether the purpose was adjustment for confounders, as no list of confounders was provided.

Level of evidence of most articles (8/11) was classified as Level 3c (i.e., cross-sectional study design) [2,10,12,13,14,15,16,32]. The remaining three articles were classified as Level 3b, prospective study design with limited population [3,4,11].

### 3.3. Injury Estimates

**Figure 2:**
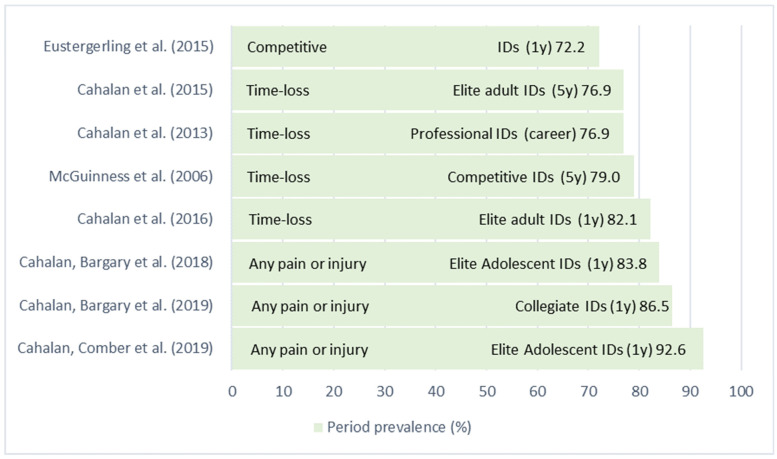
Period Prevalence by Article with Injury Definition Type, Population and Time Frame. Note. IDs = Irish dancers; y = year [3,10,11,12,13,14,15,32].

The period prevalence included one year [3,10,11,12,15], five years [13,32] and the entire career periods [14] (ranges of 72.2–92.6%) (Figure 2), with more inclusive injury definitions presenting higher prevalence. Due to the heterogeneity of populations, time frame period, and different injury definitions in the included studies, an overall summary prevalence estimate could not be calculated.

From the three included prospective studies, two provided injury incidence according to two different injury definitions. When injuries were defined as any pain or injury, incidence ranged from 9.3 to 10.6 injuries per 1000 h danced [4,11]. When injuries had a time-loss definition, injury incidence ranged from 3.4 to 4.5 injuries per 1000 h danced [4,11].

### 3.4. Anatomical Location

**Figure 3:**
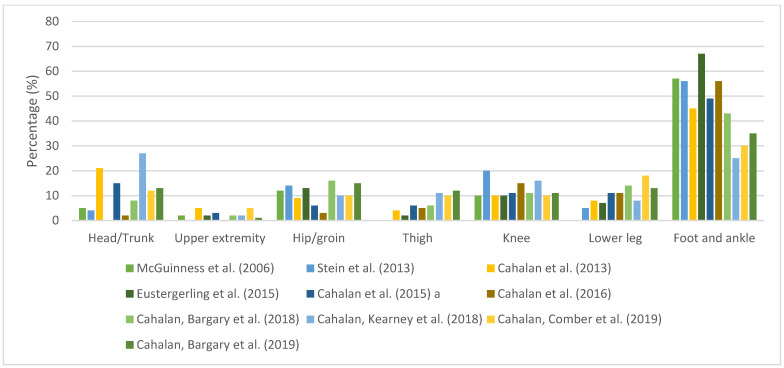
Anatomical Injury Distribution. Note: One article [2] is not included because classification does not fit in presented categories. Percentages do not sum to 100%. Only main anatomical locations are displayed. ^a^ In the referred article, as hip or thigh corresponded to 9.4%, this percentage was evenly divided by the categories of hip/groin and thigh for this graphic [3,4,10,11,12,13,14,15,16,32].

Detailed anatomical location by each article is available in Table S1. The foot and ankle are the most affected body areas (25–68%). Knee accounted for 9.6–20% of injuries, followed by head/trunk (0–33.9%), lower leg (0–18.4%), hip/groin (2–14%), and thigh (0–12.3%). Upper extremity comprised 0–5% of all injuries. Due to a different injury anatomical location classification across studies, anatomical locations were grouped into broader categories as presented in Figure 3. These categories of body regions were based on the International Olympic Committee consensus statement recommendations [20] along with the available information from the included studies.

### 3.5. Type of Injuries

**Figure 4:**
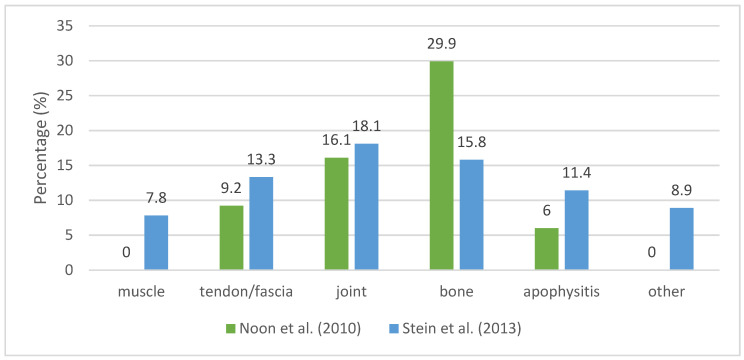
The Distribution (%) of Injury Types Reported in Medical Records [2,16].

In studies where data source were medical records (Figure 4), Noon et al. [2] found stress fractures to be the most prevalent injury (29.9% of total injuries), affecting sesamoids, metatarsals, navicular, first proximal phalanx, and tibia. Patellofemoral pain syndrome (PFPS), Sever’s disease, ankle sprains, posterior tibialis tendonitis, and plantar fasciitis were the following most frequent diagnoses.

Stein et al. [16] reported that the most frequent types of injuries were tendon injury, apophysitis, patella pain or instability, stress injury (including medial tibial stress syndrome, stress reaction, and stress fracture), muscle injury, ligament injury and fracture (excluding stress fractures).

**Figure 5:**
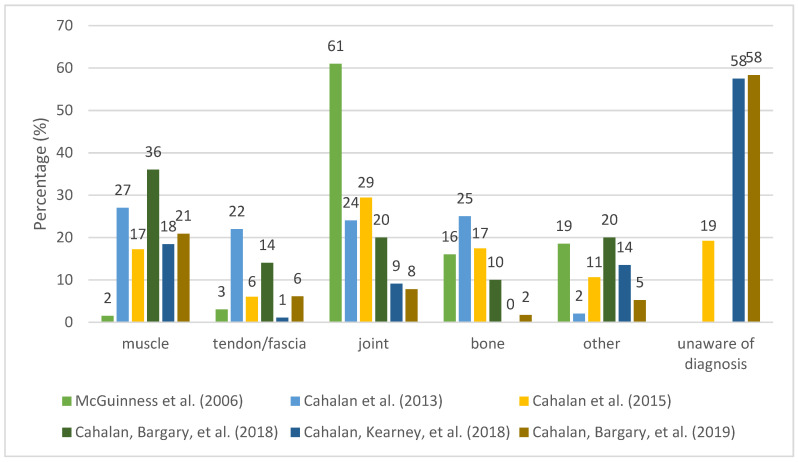
The Percentage Distribution of Self-Reported Injury Types [4,10,11,13,16,32].

From the studies where data were self-reported (Figure 5), the most common type of injury was joint injuries, followed by muscle injuries and bone injuries. Frequently reported injuries in more than one study (self-reported injuries) were muscle strains, plantar fasciitis, tendinopathies, ankle sprains, stress fractures, patellofemoral pain, apophysitis and shin splints.

### 3.6. Nature, Severity and Aetiology of Injuries

Only three studies reported the nature of injuries. Noon et al. [2] stated that the majority of injuries were overuse without providing magnitudes, Stein et al. [16] reported that 79.6% of injuries were overuse and 20.4% were acute or traumatic, and Cahalan et al. [12] reported the injury onset, where 33.3% were sudden onset, 36.4% gradual onset and 30.3% unsure or not stated.

Severity definitions, when provided, were all based on time loss from dance performance and rehearsal. Most injuries were mild or moderate except in one study [13]. Mechanism of injury was not described in detail in any of the included studies. McGuinness and Doody [32] found that most injuries occurred before a major competition (58%) or when new steps were introduced (21%). In professional Irish dancers [14], 49.2% were injured mid-way through a production run and 10.8% became injured in the rehearsal period of the production run. The most serious injuries occurred near the end of tour (20.8%) and in the early stages of touring (20%). 

### 3.7. Associated Factors 

Results of analysed factors and statistical approach are summarised in Table 3, Table 4, Table 5, Table 6 and Table 7.

## 4. Discussion

This systematic review highlights the high number of injuries affecting Irish dancers, mostly in the foot and ankle, with an overuse nature. Also, some risk factors were identified, and the analysis of the included study methodologies led to recommendations for future studies. 

### 4.1. Summary of Evidence 

#### 4.1.1. Prevalence/Incidence

Self-reported injury prevalence is higher in Irish dancers than in preprofessional [33] and professional ballet dancers [34] and tap dancers [35]. However, the included studies used different injury definition, population, and time frame period. The low prevalence (59%) of self-reported injuries (time-loss definition) in tap dancers [35] compared to Irish dancers might occur because tap dance steps generate lower ground reaction forces (mean 2.06 ± 0.55 body weight), joint forces, and moments compared to Irish dance steps, whereas in Irish dance, high ground reaction forces have been observed [36,37,38], as well as individual peak forces reaching up to 9.86 times the body weight [38]. 

Three factors might underestimate injury prevalence in the included studies: (i) a large recall period in retrospective studies that may lead to errors of omission as minor injuries tend to be forgotten [39], (ii) time-loss injury definition [33,40,41], (iii) dancers not reporting all their injuries or complains and dancing through pain. This may be partially explained due to fear of stopping dancing and a culture of injury/pain concealment, perseverance, and accepting or ignoring injury as well as the belief that pain is an inherent part of dancing [10,34,42,43].

The injury incidence was reported only in two studies [4,11] with different age group samples and using small samples (n = 21 and n = 37), which make it difficult to draw any conclusions. Both studies report injury incidence higher than other dance styles [33,44,45,46,47,48] when using a non-time-loss injury definition, although when using a time-loss definition, the incidence falls within the same values for ballet [49,50]. 

#### 4.1.2. Occurrence Pattern of Musculoskeletal Injuries

The higher number of injuries happened in the lower extremity (LE), where the foot and ankle are the most affected anatomical structures. Irish dance puts a tremendous demand on the LE, particularly in the foot–ankle complex [36,37,38]. Its explosive work and the associated effect of fatigue and subsequently increased anterior shear and compressive forces in the ankle [51], as well as the landing strategy used in Irish dance (e.g., extended knees and plantar flexed ankles) may play a key role in exposing the foot–ankle complex to repetitive forces. For instance, Irish dance technique requires an extended landing posture and dancing constantly on toe tips and metatarsal heads, with repetitive forefoot push off [7,31]. Contrastingly, ballet dancers have a landing strategy adequate for better shock absorption: increased forefoot strategy at initial contact followed by an increased range of motion at the ankle and knee joints [52]. This likely prevents Irish dancers from attenuating ground reaction forces, as the foot–ankle complex excursion contributes to mitigate the landing forces [53,54] as well as the hip and knee flexion [55,56]. 

Two studies report a high proportion of bone injuries, in particular stress fractures (29.9% of total injuries) [2] and stress injuries (10.1% of total injuries) [16] reported by healthcare professionals. This difference might be due to different sample characteristics, diagnostic methods (magnetic resonance imaging, clinical), and level/intensity of training. Moreover, Noon et al. [2] mention that most dancers (79.7%) had multiple recorded injuries, and a repeat injury was counted as a second injury, which might over-represent the proportion of stress fractures. While in one study the sample was composed of female dancers from a single Irish dance company (n = 69) [2], the other was composed of 255 dancers from 37 different schools [16]; therefore, the study may be more representative.

Stress fractures, as an overuse injury, result from submaximal, repetitive loading [57]. Noon et al. [2] relates the high proportion of sesamoid stress fractures to repetitive stamping, frequent jumps and high-impact landings and compensatory turnout (foot pronation and metatarsophalangeal joint valgus rather than external hip rotation). Indeed, activities with repeated submaximal stresses and new or excessive exercise patterns with limited rest are risk factors for stress fractures [58].

Footwear and hard surface training are also potential risk factors for stress fractures [59]. It was found that the different footwear worn by Irish dancers exert significantly different forces on the dancers’ feet, particularly in the forefoot region, with soft shoe resulting in higher plantar loads compared to hard shoe and dance trainer [60]. Also, it was observed that split sole sneakers (also known as dance trainers) were associated with a decreased incidence of injury in Irish dancers [32]. The frequent time spent dancing on soft shoes, plus dancing on a hard surface, along with the repetitive movements with considerable impact, without “plié” on landing, may indicate that excessive external training load contributes to stress fractures in Irish dancers.

Patella pain or instability and PFPS accounted for 10.8% and 11% of injuries [2,16]. In a retrospective chart review study characterising knee injury pattern in young Irish dancers, the most frequent diagnoses (53.5% of knee injuries) were related to patellar tracking disorders, including PFPS, hypermobile patella, and patellar sub-luxation [29]. In ballet, the effects of forcing lower extremity turnout are well documented through compensatory strategies elsewhere along the kinetic chain, leading to feet pronation, external tibial torsion, valgus knee stress, increased Q angle, and lateral patellar tracking [61,62]. As turned-out lower extremities are a common characteristic of Irish dance and ballet, they could be implicated in patellar tracking disorders in Irish dancers. 

Also, Irish dance repetitive movements with considerable load might contribute to overuse injuries, in particular PFPS, especially when fatigued, with Irish dancers presenting a significantly greater external knee flexion moments in landing [51]. In addition, Irish dancers can have a demanding calendar of practice and competition, with irregular spikes and dips in dance exposure [4,10]. Thus, it is paramount to monitor and manage workload, to ensure gradual adaptation to the required loads, prevent spikes in loads, and to enhance physical qualities [63]. Moreover, periodisation of training should be implemented for prevention of overtraining, underperformance, and increased injury occurrence [64].

The six included studies describing self-reported injury type have considerable variability, as the data were provided by dancers that may or may not have a diagnosis from a licensed healthcare practitioner. This means that data depended on recall (in retrospective surveys) and could not always warrant an accurate or valid diagnosis, thus raising questions about the validity of these results [17], especially taking into account that injury diagnosis is not very reliably recalled [65]. Three articles have long recall periods, making them more vulnerable to effects of recall bias [13,14,32]. In one study, participants were asked to recall the three most recent injuries in the previous five years [32], which potentially resulted in dancers reporting injuries that were the most serious to them, and missing those with the least impact. Also, the mentioned six studies did not use a validated questionnaire, or it was not possible to determine the validity and reliability of the questionnaire to obtain injury type data. 

Considering all the mentioned methodological limitations, results show a trend of muscle and joint to be the most affected by injury, followed by bone. It should be noted that injury types presented in the category of “other (not discriminated)” represent a considerable proportion. This may possibly influence the observed results, further limiting their interpretation. In addition, two articles are part of the same study, with the same sample but different study design: one prospective [11], and the other cross-sectional [10]. Three studies report that a large proportion of dancers were unaware of diagnosis [4,11,13], which further limits the possibility to draw conclusions about injury type. 

None of included articles described the mechanism of injury in detail. The nature of injuries was reported in only three studies: mostly overuse [2,16] or with a gradual onset [12]. Despite the fact that the nature of injuries is not reported in other included studies, it can be hypothesised that the types of injuries are mostly overuse in nature. Although the injury mechanism is not described, frequently reported injuries in the included studies (e.g., plantar fasciitis, tendinopathies, stress fractures, PFPS, apophysitis and shin splints) are injuries usually caused by repetitive microtrauma, thus with more potential for prevention. 

Irish and ballet dances are highly demanding and the nature of injury in both is predominantly overuse [47,66]. Together with insufficient physical preparation, poor planning of training/rehearsals [28], faulty technical execution [67], repetitive movements without adequate rest [68], competitive environment, perfectionist mindsets, and consequent psychosocial distress [69,70], and the dancers’ increased pain tolerance [71] can make dancers vulnerable to overuse injuries. Additionally, it seems that Irish dancers do not benefit from the landing technique used in ballet to mitigate the landing forces, most likely aggravating the risk for overuse injuries in the LEs. 

For development of injury prevention programs, it is essential to better understand the causes of an injury in a specific context and the risk factors [72,73]. Given the complexity of mechanisms precipitating an injury, especially overuse injuries, multidisciplinary approaches and a combination of different research approaches are needed [73,74]. 

Cahalan and colleagues [3,4,11,13,14] provide an insight into Irish dancer’s perceived causes of injuries. The most frequently mentioned self-perceived cause of injury is overuse and fatigue, which is in accordance with the literature regarding dance [75,76] and circus [77]. This is an important clue that training load should be monitored and managed, as it is already practiced in the sports field [78,79,80].

The severity of most injuries appears to be mild, although that is not clear. Studies used a time-loss severity definition, which has some limitations. First, dancers may be able to participate before an injury has fully resolved with technique adaptations [20], or they can “soldier on” with their injury [14], or injuries might not result in abstention from dance [4]. Second, many injuries are of gradual onset, chronic or recurrent, not reflecting the true severity, as dancers can have low time loss but be affected in content and intensity of training/performance. The above-mentioned aspects tend to cause underestimation of the absolute severity of injuries if one considers full healing as the gold standard [20].

#### 4.1.3. Associated Factors

Most included studies analysing risk factors had a cross-sectional design and/or only reported associations or used univariate tests. This approach is not adequate for the multifactorial nature of sports/dance injuries and does not address the complex interactions among several risk factors. Nevertheless, these studies provide data to guide the identification of risk factors that can be analysed in longitudinal studies, with analytical approaches that incorporate repeated measures (monitoring variables over time) and using a multivariate statistical approach.

Regarding demographic factors, only two studies examined the relation between gender and injury, with conflicting results. Age also remains inconclusive, but there is a trend toward more injuries with increasing age. In ballet dancers, evidence about age as a risk factor is inconclusive as well [27,28]. Although gender and age are non-modifiable factors, it is important to know if they can influence other variables, so they can be adjusted for confounding. 

In the included studies examining dance exposure, results do not suggest a relation to injury. These data were collected via retrospective questionnaires, with possible errors associated to recall, and there was a great variability in the cohort regarding: the number of hours/week spent at dancing classes, the number of hours/week spent practicing at home, and the number of competitions/year [32]. The number of participants in the prospective cohort study was small (n = 27), and dance exposure was self-reported [4]. 

On the other hand, in the prospective study with elite adolescent Irish dancers, increased weekly hours of dance training were associated with a decreased total number of weeks injured, suggesting dance load as a protective factor [11]. In this cohort, weekly dance exposure was also low (mean = 7.9 h/week) in comparison to ballet. In an elite-level ballet company, dancers can perform a mean of 34 h per week [50], and pre-professional ballet dancers can perform an average of 30.3 h per week [81]. 

Dance exposure has been identified as a possible risk factor in ballet and contemporary dancers, although evidence remains inconclusive [27]. Although there is evidence of a relationship between training load and injury in competitive athletes [82], load as a single factor does not explain nor predict all injuries [83] because of the complexity and multifactorial nature of injury aetiology [84]. Load could be analysed coupled with other variables to identify dynamic interactions [83,84]. Two of the included studies examining dance exposure simply compared means [4,32], and one used statistical tests that assume linearity of relationships [11]. 

Also, the included studies only used an external measure of training load (dance hours/week). Hours per week of dance is likely not the best external load measure for comparison with other dance styles because of the ballistic and explosive nature of Irish dance. 

Only one study investigated the impact of physical growth on injury in elite Irish dancers. This prospective study found no relationship between total number of injuries or the total number of weeks injured and change in weight or height [11]. In elite adolescent ballet dancers, it was found that rate of growth (foot length change) is likely associated with a small to moderate increase in risk of lumbar and lower extremity overuse injury [85]. Since apophysitis is frequently reported in the included studies, it would be plausible to find a relation between rate of growth and injury in Irish dancers. Some possible explanations are the different methodologies to access growth as well as the age of the included study cohort (aged 13–17) and the median growth in height (3, IQR = 2, 7 cm), which may reflect that most of these dancers were not in the period around the peak height velocity when there is an increased risk of injuries in elite sporting populations [86,87,88]. Also, the dance exposure in the Irish dance cohort was low (mean = 7.9 h/week), and most participants reported their sleep quality as being good or very good [11].

Irish dancers competing at a higher level seem to be at a higher risk of injury. One study found that dancers at an elite level were more likely to report an injury than dancers who were non-elite (OR = 6.33, CI 1.27–31.57) [15]. Additionally, it was observed that the average number of injuries per dancer increased as the dancer’s skill level increased [2]. This might be related to the increased dance exposure of higher levels, the more complex and demanding choreography, as well as the greater competitiveness and related psychosocial stressors. Conversely, years of participation in Irish dance do not appear to relate with injury. 

Cross-training was examined as possibly related to lower injury rates in four studies, but no significant correlation was found [3,13,14,15]. None of the studies provide the components of cross-training activities reported, and dancers undertook little weekly cross-training or none at all [10,11,14].

Performing a warm-up and cool-down was associated with a decreased incidence of ankle injury [32]. In a prospective study, failing to always perform a warm-up was significantly associated (*p* = 0.042) with foot and ankle pain and injury in elite adult Irish dancers [30]. Two studies found no significant correlation with injury, but there was a great compliance with warm-up and cool-down in this cohort [14], which makes it difficult to assess their impact on injury, and the proportion of dancers who perform a warm-up or cool-down was not provided [15]. Also, none of referred studies described in detail the components of warm-up or cool-down. Therefore, despite the limitations of included studies, performing a warm-up and cool-down is likely an important factor for injury prevention.

None of the three included studies examining physical factors found significant associations with pain/injury. This is conflicting with the evidence regarding ballet dancers, where several physical factors were identified, such as anthropometrics, poor aerobic capacity, hypermobility, degree of turnout, core and LE weakness, and range of motion discrepancies of the LE [27,28,89,90]. 

First, two articles reported data from the same study (one is cross-sectional and the other is prospective), and physical variables were within normal values [3,13]. Also, in the prospective study, physical variables were measured only at the baseline, which does not account for temporality [91], as they could change over time. 

Second, some physical variables measured might not be sensible or adequate for the Irish dance population. For example, the Movement Competency Screen includes movements that might not be relevant for Irish dancers, such as the push-up, because Irish dancers keep their arms close to the torso while dancing and the injury proportion of the upper extremities is negligible. Also, physical endurance was measured with plank [10], which does not address other important muscle groups. For instance, calf endurance would likely be important to access, since Irish dancers stand on toes most of the time (standing on the forefoot with ankle plantar flexion) and repeatedly jump and land on toes as well. 

Third, injury aetiology is complex and multifactorial [84,92], and it is unlikely that a single physical factor can predict injury. The included studies examining physical factors focused on the contribution of isolated factors. 

Regarding general health complains, most of included studies consistently reported an association with injury [3,4,11,13]. These results emphasise the importance of including general health complains (beyond musculoskeletal complains) in screening tools for dancers. 

From the strongest level of evidence in the included studies (i.e., prospective cohort design), findings are in accordance with the literature [93,94,95] as insufficient sleep was associated with more pain/injury [3], and participants with better sleep had fewer days lost due to injury [4]. In a different cohort, there was no association with sleep quality and injury, but most dancers reported good or very good sleep quality [11]. 

Psychosocial factors were moderately represented in the included studies, with only Cahalan and co-workers examining these factors. The number of psychological health complains or problems was significantly associated with injury [13,14] or close to statistical significance (*p* = 0.054) [3]. 

It was also identified that psychological distress was common among professional Irish dancers, with “general anxiety”, “tension with other people”, “stress due to external factors”, “performance anxiety”, and “overuse of drugs or alcohol”, frequently reported when working as a professional Irish dancer [14]. In elite adult Irish dancers, it was found that scoring poorly on two subscales of the Athletic Coping Skills Inventory (coping with adversity, and goal setting and mental preparation) was significantly associated with foot and ankle pain and injury [30].

In elite adolescent athletes, self-esteem is a risk factor for injury [96], assessed by the Competence-Based Self-Esteem Scale, which describes contingent self-esteem dependent on competence as well as self-criticism and feeling of insufficiency [97]. Most Irish dancers participate in competition-based activity [43], and it has been observed that some competitive Irish dancers are ego-motivated [12], possibly experiencing shame and loss of self-esteem when they cannot achieve expected results [98]. 

Despite all the evidence about the importance of psychosocial factors in injury risk and recommendations to include them in screening [99] as well as in injury epidemiology research [72,84], non-physical factors still need to be better integrated [17,100]. A better knowledge of psychological, social and contextual factors is crucial not only for studying injury aetiology but also for injury recovery, and for risk factor management in injury prevention strategies [43,101,102,103]. Furthermore, Irish dancers rarely point out psychological factors as perceived causes of injuries [3,4,11,13,14], and therefore education might play a relevant role to increase awareness.

Overall, the associated factors identified in this systematic review require an in-depth investigation due to the heterogeneity of factors assessed, different methods, and low level of evidence of the included studies (mostly cross-sectional). Moreover, studies with prospective design did not use repeated measures, and one prospective study did not measure exposure, preventing us from calculating incidence and risk.

None of included studies clearly identified potential confounders. Despite the fact that it is debatable whether confounding should be addressed only in studies with causal questions [104,105], we argue that in studies investigating non-causal associations the role of additional variables should at least be identified/anticipated and analysed, for example, with subgroup stratification or using a multivariate statistical approach [106,107]. This would help the reasoning for designing studies investigating why dancers sustain injuries, providing evidence for the development of intervention programs for injury prevention.

Based on the findings of this systematic review, a summary of recommendations for future research is presented in Box 1, as well as recommendations for health professionals (Box 2).

Box 1 **Recommendations for Future Research.**

- Provide a clear injury definition.
- Measure exposure accurately and with valid and reliable methods.
- Differentiate recurrent from new injuries.
- Describe, when possible, the mechanism of injury in detail, such as the dancer situation (context), behaviour, and biomechanical descriptions.
- Limit the recall period to 6/12 months if using retrospective questionnaires.
- Base the data collected on body area, and symptoms rather than specific diagnosis, if injury data is self-reported.
- Report the type of injury via healthcare professional, with as much detail as possible, combining injury region, type, and diagnosis.
- Complement time-loss and medical attention injury measures with a valid and sensitive self-report instrument.
- Examine risk factors prospectively, with analytical approaches that incorporate repeated measures and using a multivariate statistical approach.
- Identify and control potential confounders.
- Investigate what type, duration and intensity of warm-up and cool-down would be most beneficial for Irish dancers.
- Analyse psychosocial variables, such as catastrophizing, mood, self-esteem, and coping skills, when examining risk factors.
- Measure internal and external loads. Utilise non-linear modelling techniques when examining the relationship between training load and injury.
- Address growth-related risk factors and maturational status in young Irish dancers.

Box 2 **Recommendations for Health Professionals.**

- Injury surveillance should be implemented in schools and companies. It would allow an ongoing and systematic collection and analysis of data.
- Injury surveillance should include (not exclusively) sleep quality assessment; general health assessment; a self-report instrument sensitive to overuse injuries; self-report measures of mood, catastrophizing, and coping skills; physical fitness tests of muscular strength, power, balance, flexibility, and endurance.
- Health professionals should be aware of most frequent types of injuries in Irish dancers to better understand and manage the potential risk factors.
- Preventive interventions should focus (although not exclusively) on lower extremities, especially foot and ankle, as they are the most affected body areas.
- Irish dancers should be knowledgeable of psychosocial factors associated with injury.

### 4.2. Limitations

This systematic review has some limitations which are related to methodological aspects and level of evidence of the included studies. Heterogeneity in injury definition, outcome measures, and methods of data collection hampers comparison. Four studies did not report a clear injury definition, compromising validity. Most studies used retrospective questionnaires, and three had a recall period over one year, which makes them prone to recall bias [65]. Many dancers did not have an injury diagnosis, and the accuracy of self-reported injury is highly questionable [17]. Also, in most studies, it was not possible to determine the validity and reliability of the questionnaire to obtain injury type data. Female dancers were 5.7 times more represented in included studies; thus, the results may not be generalisable to male dancers.

Studies analysing associated factors did not control for potential confounders, limiting the accuracy of results [108]. Also, most included studies did not assess potential interaction of risk factors, and none used repeated measures. Most studies were conducted by the same authors, and three studies resulted in six articles, decreasing the overall sample size. Injury recurrence and injury mechanism were not fully explored in most of the included studies.

In this systematic review, only studies written in English or Portuguese language were included, published in peer-reviewed scientific journals, to assure quality and validity.

## 5. Conclusions

In the present systematic review, the high number of injuries affecting Irish dancers is notorious, mostly in the foot and ankle, which is likely explained by the great demands placed on these functional units. Bone or muscle/joint injuries were the most common type, depending on the reported data method, and psychological factors, elite level, failure to complete a warm-up, health complains, and insufficient/poor sleep were associated with injury. Considering that Irish dance is such a demanding dance style, especially for the LE, and that studies of high level of evidence are still lacking, more studies are needed to improve performance and reduce injuries in this growing population of dancers.

Quality of studies must be improved by providing a clear injury definition, use of validated tools, and measuring exposure, to allow for calculating incidence and risk. Studies examining risk factors should use a methodology that considers the multifactorial nature of dance injuries and the complex interactions among risk factors. Future studies should use a prospective study design with analytical approaches that incorporate repeated measures and use a multivariate statistical approach. An effort should be made to identify and control for confounders.

Although some advances in Irish dance injury epidemiology were made, high-quality studies to allow deep knowledge in this area to enable the development and implementation of effective injury prevention and intervention programmes are still lacking. Because of the high levels of injury in Irish dancers and the evolution to an athletic style, Irish dance schools and companies would benefit from an injury surveillance system.

## Figures and Tables

**Table 1:** Study Design, Data Source and Participant Details for Included Studies.

Author (Year)	Study Design (Data Source)	Sample Size (n=)	Age (Means ± SD (Range) Years	Sex (F/M)	Level	Dance Experience (Means ± SD Years)
McGuinness et al. (2006) [32]	Cross-sectional retrospective (questionnaire 5 y)	159	18 ± 3 (15–27)	142/17	Competitors	11 ± 4 (1–20)
Noon et al. (2010) [2]	Cross-sectional retrospective (chart review 7 y)	69	13.1 (8–23)	69/0	3 (compete in small, local competitions), 4 (as well as regional competitions) and 5 (qualify for international competitions)	Not reported
Stein et al. (2013) [16]	Cross-sectional retrospective (medical records 11 y) + Cross-sectional retrospective (questionnaire)	255	13.7 ± 5 (4–47), 95% < 19	247/8	Not reported	Not reported
Cahalan et al. (2013) [14]	Cross-sectional (retrospective online questionnaire—entire career)	178	>18.72% (25–34)	111/67	Professional	13.2 ± 3.4
Eustergerling et al. (2015) [15]	Cross-sectional retrospective questionnaire (1 y)	36	16 (12–46)	35/1	22 elite/14 non-elite	Not reported
Cahalan et al. (2015) [13] Cahalan et al. (2016) [3]	Cross-sectional retrospective (questionnaire 5 y + physical screening)	104 (questionnaire) 84 (physical screening)	Median (IQR) prof. 23 (21; 27.5), stud. 20 (19.2), comp. 20 (18.5; 20)	% prof. 50/50 stud. 85.7/14.3 comp. 80/20	Elite: 36 (34.6%) professionals, 28 (26.9%) students, 40 (38.5%) competitive	Prof. 17.5 ± 5.6 stud. 13.8 ± 5.3 comp. 13.0 ± 3.8
	Prospective cohort (1 y) (online questionnaire every month)	84	Median (IQR) 20 (19–23.5)	66/18	Elite: 15 professionals, 31 students, 38 competitive	14 (approx.)
Cahalan, Kearney et al. (2018) [4] Cahalan, Comber et al. (2019) [12]	Prospective (questionnaire every week over 1 year)	21 ID + 29 CD	21.5 ± 1.7	20/1	Pre-professional students (full-time students in university)	“Extensive dance experience”
	Cross-sectional (retrospective questionnaire 1 y + physical screening)	27 ID	Median (IQR) 21(3)	24/3	Pre-professional students (full time students university)	“Extensive dance experience”
Cahalan, Bargary et al. (2019) [11] Cahalan, Bargary et al. (2018) [10]	Prospective (questionnaire every week over 1 year)	37	13–17	33/4	Elite	Competing at open (elite) level for a period of at least 1 year
	Cross-sectional (retrospective questionnaire 1 y + physical screening)	37	13–17	33/4	Elite	Competing at open (elite) level for a period of at least 1 year

Note. ID = Irish Dancers; CD = Contemporary Dancers; IQR = interquartile range.

**Table 2:** Modified Downs and Black Quality Assessment Tool Scores and Level of Evidence.

Items/ Reference	McGuinness et al. (2006) [32]	Noon et al. (2010) [2]	Stein et al. (2013) [16]	Cahalan et al. (2013) [14]	Eustergerling et al. (2015) [15]	Cahalan et al. (2015) [13]	Cahalan et al. (2016) [3]	Cahalan, Bargary et al. (2018) [10]	Cahalan, Kearney et al. (2018) [4]	Cahalan, Comber et al. (2019) [12]	Cahalan, Bargary et al. (2019) [11]
**Reporting**											
**1**	1	1	1	1	1	1	1	1	1	1	1
**2**	1	0 ^a^	1	1	0 ^a^	1	1	0 ^a^	1	0 ^a^	1
**3**	1	1	1	1	1	1	1	1	1	1	1
**5**	0	0	0	0	0	0	0	0	0	NA	0
**6**	1	1	1	1	1	1	1	1	1	1	1
**7**	1	0	0	1	1	1	1	1	1	1	1
**10**	1	0	1	1	0	1	1	1	1	1	1
**External** **Validity**											
**11**	0	0	0	0	1	0	0	0	0	0	0
**12**	0	0	0	0	0	0	0	0	0	0	0
**Internal** **Validity (Bias)**											
**16**	1	1	1	1	1	1	1	1	1	1	1
**18**	1	0	1	1	1	1	1	1	1	1	1
**20**	0	1	1	0	1	1	1	1	1	1	1
**Internal Validity** **(Confounding)**											
**21**	1	1	1	1	1	1	1	1	1	NA	1
**22**	1	1	1	1	1	1	1	1	1	NA	1
**25**	0	0	0	0	0	0	0	0	0	0	0
**Score**	10	7	10	10	10	11	11	10	11	8	11
**Percentage Score (%)**	63	44	63	63	63	69	69	63	69	67	69
**Level of Evidence**	3c	3c	3c	3c	3c	3c	3b	3c	3b	3c	3b

Note. NA: not applicable. ^a^ No injury definition.

**Table 3:** Training-Related Factors and Statistical Approach.

	Authors	McGuinness et al. (2006) [32]	Cahalan et al. (2013) [14]	Eustergerling et al. (2015) [15]	Cahalan et al. (2015) [13]	Cahalan et al. (2016) [3]	Cahalan, Bargary et al. (2018) [10]	Cahalan, Kearney et al. (2018) [4]	Cahalan, Bargary et al. (2019) [11]
Factors									
Use of split sole sneakers		↓							
Cool down		↓	=	=					
Warm up		↓		=	↑ failure to complete				
Cross-training			=	=	=	=	=		
Dance exposure		=					=	=	↓
Statistics		Univariate	Univariate	Odds ratios (association)	Univariate, multivariate	Multivariate	Univariate	Univariate	Multivariate
Level of evidence		3c	3c	3c	3c	3b	3c	3b	3b

Note. = not significant; ↑ risk factor (correlated with injury); ↓ protective factor (negatively correlated with injury).

**Table 4:** Individual Factors and Statistical Approach.

	Authors	McGuinness et al. (2006) [32]	Cahalan et al. (2013) [14]	Eustergerling et al. (2015) [15]	Cahalan et al. (2015) [13]	Cahalan et al. (2016) [3]
Factors						
Elite level				↑		
Years dancing/experience		=	↑	=	=	↑
Sex			=		↑ ♀	
Age		=	↑	↑	=	
Statistics		Univariate	Univariate	Odds ratios (association)	Univariate, multivariate	Multivariate
Level of evidence		3c	3c	3c	3c	3b

Note. = not significant; ↑ risk factor (correlated with injury); ♀ female sex.

**Table 5:** Psychological Factors and Statistical Approach.

	Authors	McGuinness et al. (2006) [32]	Cahalan et al. (2013) [14]	Eustergerling et al. (2015) [15]	Cahalan et al. (2015) [13]	Cahalan et al. (2016) [3]	Cahalan, Bargary et al. (2018) [10]
Factors							
Psychological problems/complains			↑		↑	=	
Lower mood					↑	=	=
Higher catastrophizing					↑	=	=
Higher levels of anger-hostility					=	↑	↑
Statistics		Univariate	Univariate	Odds ratios (association)	Univariate, multivariate	Multivariate	Univariate
Level of evidence		3c	3c	3c	3c	3b	3c

Note. = not significant; ↑ risk factor (correlated with injury).

**Table 6:** General Health and Sleep-Related Factors and Statistical Approach.

	Authors	Cahalan et al. (2013) [14]	Cahalan et al. (2015) [13]	Cahalan et al. (2016) [3]	Cahalan, Bargary et al. (2018) [10]	Cahalan, Kearney et al. (2018) [4]	Cahalan, Bargary et al. (2019) [11]
Factors							
Higher number of subjective health complaints			↑	↑		=	
A higher level of general everyday pain				↑			
More body parts affected by pain/injury			↑	↑	↑		
Always/often dancing in pain		=		↑	↑		
Insufficient/poor sleep				↑		↓ better sleep	=
General health scores						↓	
Number of weeks participants reported poor/very poor general health							↑
Statistics		Univariate	Univariate, multivariate	Multivariate	Univariate	Univariate	Multivariate
Level of evidence		3c	3c	3b	3c	3b	3b

Note. = not significant; ↑ risk factor (correlated with injury); ↓ protective factor (negatively correlated with injury).

**Table 7:** Physical Factors and Statistical Approach.

	Authors	Cahalan et al. (2015) [13]	Cahalan et al. (2016) [3]	Cahalan, Bargary et al. (2018) [10]	Cahalan, Bargary et al. (2019) [11]
Factors					
Physical screening tests		=	=	=	
Change in weight or height					=
Statistics		Univariate multivariate	Multivariate	Univariate	Multivariate
Level of evidence		3c	3b	3c	3b

Note. = not significant.

## Data Availability

The data can be requested from the authors.

## Supplementary Materials

The following supporting information can be downloaded at: https://www.mdpi.com/article/10.3390/ijerph20126190/s1, Table S1: Consensus Data Extracted.

## Appendix A

Data Extraction Form

**Article title:**
**Sample size:**
**Participant details (age/gender/others):**
**Study purpose:**
**Dance experience:**
**Study design:**
**Injury definition:**
**Outcome measures:**
**Inclusion/exclusion criteria:**
**Number of injuries:**
**Injury estimates (incidence, prevalence, time exposure):**
**Nature of injuries (acute traumatic vs chronic overuse):**
**Anatomic injury sites:**
**Types of injuries/symptoms:**
**Injury’s severity:**
**Most common injuries:**
**Associated risk factors:**
**Main limitations & recommendations:**
**Aetiology (mechanism of injury, inciting events):**
**Comments:**
