# Supplementary material for: Irish Dancing Injuries and Associated Risk Factors: A Systematic Review
Source: Int J Environ Res Public Health. 2023 Jun 20;20(12):6190. doi: 10.3390/ijerph20126190 (PMC10298157; doi:10.3390/ijerph20126190)
Supplement: Supplementary file 1 [file ijerph-20-06190-s001.zip › ijerph-2348583-supplementary.pdf]

Table S1. Consensus Data Extracted.

	**McGuinness et al. (2006)**	**Noon et al. (2010)**	**Stein et al. (2013)**	**Cahalan et al. (2013)**	**Eustergerling et al. (2015)**	**Cahalan et al. (2015)**	**Cahalan et al. (2016)**	**Cahalan, Bargary et al. (2018)**	**Cahalan, Kearney et al. (2018)**	**Cahalan, Comber et al. (2019)**	**Cahalan, Bargary et al. (2019)**
**Article title**	The Injuries of Competitive Irish Dancers	Injury Patterns in Female Irish Dancers	Injuries in Irish Dance	Injury in Professional Irish Dancers	Risk Factors for Injuries in Competitive Irish Dancers Enrolled in Dance Schools in Calgary, Canada	A Cross-Sectional Study of Elite Adult Irish Dancers Biopsychosocial Traits, Pain, and Injury	Inability to perform because of pain/injury in elite adult Irish dance: A prospective investigation of contributing factors	Pain and Injury in Elite Adolescent Irish Dancers: A Cross-Sectional Study	Dance exposure, wellbeing and injury in collegiate Irish and contemporary dancers: A prospective study	Biopsychosocial Characteristics of Contemporary and Irish University-Level Student Dancers A Pilot Study	Dance exposure, general health, sleep and injury in elite adolescent Irish dancers: A prospective study
**Sample size**	n= 159	n= 69	n=255	n=178	n=36	104 subjects completed a questionnaire, of whom 84 underwent a physical assessment.	subjetive data (n = 85); physical data (n = 84)	n=37	IDs n=21 vs contemporary dancers n=29	n= 30 contemporary dance (CD) and n=27 Irish dance (ID)	n=37
**Participant details (age/gender/others)**	*15-27 yrs (mean=17.8±2.9); 142 females and 17 males; 48% of the dancers came from USA, 21% from Canada, 15% from Ireland, 16% from England, Australia, New Zealand. Nº of years dancing ranged from 1 to 20 years (mean: 10.8; SD: 3.8). Student IDs (i.e., none of the subjects were professional dancers) and participants at the North	69 female ID who presented at an academic sports medicine centre from June 2002 to September 2009 (7 yrs). Ages 8 to 23 yrs. The mean age of the dancers was 14.3, 13.7, and 12.7 years for levels 3, 4, and 5, respectively.	247 female (97%) / 8 male (3%) - age from 4 to 47, mean 14 ± 5, with 95% (243/255) under the age of 19	61% (111) female / 39% (67) male; 72% between 25-34 yrs; mean age becoming professional Irish dancer 18.5±3.0 y; 7.5±4.6 y employed as a professional dancer	22 were elite/14 were non-elite. The average age was 16 yrs (range 12–46) and there was only 1 male dancer	Questionnaire: 74 female, 30 male; 18 yrs or older (mean female=20, mean male=22.5); 36 prof/28 stdts/40 Comptt dancers. Physical screening: 71.2% female (21.2±3.1 yrs) and 28.8% male (23.0±4.0 yrs)	age median 20 yrs (19-23,5); female 66 / male 18. Professional-15; Students 31; Competitive-38	4 male, 33 female, aged 13 to 17 yrs	20 female, 1 male; full time students ID group mean age 21,5 yrs (1,7 yrs SD)	Age (sex): Irish dancers=21±3 yrs (3 males, 24 females)	4 male, 33 female, aged 13 to 17 yrs, competing at the highest championship level for at least the previous year, recruited from six ID schools in Ireland

	**McGuinness et al. (2006)**	**Noon et al. (2010)**	**Stein et al. (2013)**	**Cahalan et al. (2013)**	**Eustergerling et al. (2015)**	**Cahalan et al. (2015)**	**Cahalan et al. (2016)**	**Cahalan, Bargary et al. (2018)**	**Cahalan, Kearney et al. (2018)**	**Cahalan, Comber et al. (2019)**	**Cahalan, Bargary et al. (2019)**
	American ID Championships										
**Study purpose**	to determine the rate and type of injury in competitive IDs, to establish the training methods and injury prevention measures used by dancers, and to investigate any relationships between prevention measures and incidence of ankle injury.	To determine the type of ID injuries requiring evaluation and treatment by a sports medicine physician	to analyse the types of dance injuries incurred by IDs.	to establish retrospectively the rate of musculoskeletal pain and injury among current and former PIDs, how pain and injury impacted their performance, and the etiological factors perceived to be associated with these injuries	to collect information on participation in ID, injury incidence, and risk factors for injury in competitive Irish dancers 12 years or older in Calgary, Canada	to collect baseline data on the biopsychosocial characteristics and to investigate any relationships between these characteristics and the incidence of musculoskeletal pain and injury of elite adult IDs	To examine the factors that relate to absence from dancing because of MSK pain/injury in IDs, and to inform guidelines for the development of an evidence-based screening protocol	To investigate the incidence of pain and injury in elite adolescent Irish dancers and examine potential associated biopsychosocial risk factors.	to investigate dance exposure (hours of dance activity), cross–training, sleep, general health, and injury among pre-professional student dancers from ID and CD genres.	To compare biopsychosocial characteristics of contemporary dance and Irish dance university students. Also, to investigate the engagement of these dancers with medical professionals	to establish the rates of time-loss and non-time-loss injury in this cohort. Furthermore, to explore the impact of dance exposure (hours of dance activity), cross training, sleep, general health, and physical growth on injury in elite Ids
**Dance experience**	nº of yrs spent dancing ranged from 1 to 20 years (mean: 11 ± 4 years*), 71 (45%) had been dancing for 6 to 15 years	Levels 3-5 (based on skills and competition level); level 3 (10-18 yrs mean 14.3yo, 2-3h/week); level 4 (8-23 yrs mean 13.7yo, 2-8h/week); level 5 (8-20 yo mean 12.7yo, 10-18h/week)	not reported	Mean previous dance experience was 13.2 years (sd ± 3.4 years)	22 elite (competing at a championship level); 14 non-elite (non-championship level)	36 (34.6%) professionals, 28 (26.9%) students, 40 (38.5%) competitive. Mean years dancing: prof. 17.51 ± 5.61, stud. 13.75 ± 5.31, comp. 13.00 ± 3.79	Years dancing: LTA=14.37±4.4, MTA=13.20±5. Professional-15; Students 31; Competitive-38	Subjects were actively competing at open (elite) level for a period of at least 1 year	pre-professional students (full time students University); "extensive dance experience"	pre-professional students (full time students University); "extensive dance experience"	Subjects were actively competing at open (elite) level for a period of at least 1 year
**Study design**	Descriptive epidemiology study	Cross-sectional retrospective chart review	cross-sectional - chart review	Cross-Sectional Survey (retrospective)	cross-sectional study: survey	cross-sectional	Prospective cohort study	cross-sectional study	Prospective study	pilot study	Prospective study
**Injury definition**	injury was defined as any injury that lead	not reported	injury was defined as any dance-related pain or disorder that led to	Injury was defined as any physical insult	not reported	any pains or injuries that had caused absence from	Pain/injury was defined as any physical	Any pain and injury (not clear)	Injury was defined as "any pain or	No specific definition of	Injury was defined as "any pain or injury

	McGuinness et al. (2006)	Noon et al. (2010)	Stein et al. (2013)	Cahalan et al. (2013)	Eustergerling et al. (2015)	Cahalan et al. (2015)	Cahalan et al. (2016)	Cahalan, Bargary et al. (2018)	Cahalan, Kearney et al. (2018)	Cahalan, Comber et al. (2019)	Cahalan, Bargary et al. (2019)
	to absence from training and competition for two weeks or more.		evaluation in the sports medicine or general orthopaedic clinic at the investigators' institution	that caused absence from one or more rehearsal or performance days		dancing for more than 1 week over the previous 5 years	complaint that caused absence from one or more rehearsal or performance days		injury that impacted upon their ability to dance"	injury was provided	that impacted upon your ability to dance"
**Outcome measures**	Questionnaire: demographic information (dancers age, years dancing, hours spent at classes and practice per week, competitions per week, competitions per year and type of footwear), training methods (use of warm up, type of floor and dance shoes), injury history of the 3 most recent injuries (site and type of injury and injury diagnosis, and time taken to recover from injury)	Injury data based on injury diagnosis; number of injuries/dancer; number of injuries/level of dancer, age at time of injury; stress fractures diagnosed using imaging (radiograph or MRI); relationship between injury and age, menarche, skill level.	Demographic data and descriptive information (age at time of presentation, sex, body part involved, side of injury, mechanism of injury as described by the patient, time to presentation, treatments prescribed, nr of follow up visits, and diagnosis). Survey questionnaire included the name of the dance school/studio and whether the dancer was able to return to full dance participation after injury. Open-ended question about injury prevention.	Gender, current age, and details of dance background prior to becoming a professional ID; baseline psychological characteristics were established using GSES (it assesses coping strategies employed in difficult situations, with a higher score indicative of greater self-efficacy); psychological problems (throughout their career or when injured); pain; injury profile (body parts affected, severity of the injury in terms of performance and rehearsal days missed; subjects discussed further detail of the most	A questionnaire was adapted for a dance-specific population from a previously validated sport injury survey used to examine risk factors for sport injury in Calgary high school and junior high school students. Potential risk factors for injury that were identified in the questionnaire included age, competitive level, participation in other physical activities, years of participation in ID, and performing a warm-up or cool-down.	Questionnaire: Dance and activity levels (hours of practice, frequency of warm up and cool down, footwear worn, type of stage or practice floor normally available, type ad frequency of any cross-training undertaken); physical and psychological health baseline demographic data, details of any general subjective health complaints, medication taken, smoking status, levels of drug or alcohol consumption; any psychological issues experienced by subjects in the preceding month were selected from a list of common psychological ailments); Female subjects provided data on the onset and frequency of their menses and recorded any pregnancy in the	Physical and psychological health, dance and pain/injury history, and levels of participation in other athletic activities. Validated psychometric tools were used to gather information on the subjects' characteristics in areas including pain beliefs and management, coping, levels of stress, catastrophizing, mood, self-efficacy, nutrition, and sleep. Physical screen included: anthropometry measures, lower limb flexibility and power, foot pronation, single leg balance, hypermobility, endurance, pain pressure	Questionnaire: body part affected, the diagnosis (if known), whether they were able to continue to dance (either partially or fully), and the time-loss impact of the pain and injury; identification of who made the decision to "sit-out" from some or all dance practice, whether or not a qualified medical person diagnosed their pain and injury, and whether the pain and injury was new or recurring; Subjective Health Complaints Inventory (SHCI) (non-musculoskeletal items); Standardised Nordic Questionnaire (SNQ) (body	Baseline demographic, injury, and dance history data - via online questionnaire + Week follow up questionnaire: nr of Weekly hours of dance activity and cross-training, weekly general health and sleep quality (Likert scale 1-5). Dancers reported any injury sustained during the week, body part affected, diagnosis if know, duration of the problem, and how it impacted upon their ability to dance (able to dance fully, partially, or completely unable to	Three components of data collection: two self-administered online questionnaire and a physical screening. Injury questionnaire: to list all pains or injuries in the previous 12 months; for each injury: body part affected, diagnosis (if known), whether they were able to continue to dance (partially or fully), the time-loss impact (days absent from dance), the individual who made the decision to discontinue some or all dance practice (whether or not a qualified medical person), new or recurring	Questionnaire: data collected at baseline using an online self-report questionnaire to establish demographic, injury and dance history data; participants then completed a brief online questionnaire each week for one year; data of nr of weekly hours of dance activity and cross-training undertaken; weekly general health and sleep quality; injuries sustained, body part involved, perceived cause, diagnosis, duration of the injury (nr of days affected), and impact upon their ability to dance (fully able, partially able,

	McGuinness et al. (2006)	Noon et al. (2010)	Stein et al. (2013)	Cahalan et al. (2013)	Eustergerling et al. (2015)	Cahalan et al. (2015)	Cahalan et al. (2016)	Cahalan, Bargary et al. (2018)	Cahalan, Kearney et al. (2018)	Cahalan, Comber et al. (2019)	Cahalan, Bargary et al. (2019)
				serious injury (body part, specific SK structure involved, the stage of the tour when the injury occurred (during rehearsal stage, early, mid, or late-tour stage); additional cross training undertaken and interventions available to manage physical well-being; perceived causes of injury		previous 12 months; sleep quality and quantity in the prior month; psychometric tools - CSQ, POMSACSI-28, SCOFF); pain and injury (any pains or injuries that caused absence from dancing for more than 1 week over the previous 5 years - SNQ: body parts where they had experienced physical difficulties over the previous year; body area, perceived cause of injury, related diagnosis, nr of days of performance or rehearsal consequently missed over the previous 5 years). Physical screening: anthropometric measures, tissue sensitivity, gastrocnemius and hamstrings flexibility, subtalar pronation, balance, GJH, cardiovascular fitness and endurance, functional tests, lower limb power.	thresholds, and several functional movements (squat and bilateral lunge). Differences in the baseline demographic, psychosocial, and physical variables between the MTA and LTA groups	map); Coping Strategies Questionnaire (CSQ)—Catastrophising subscale; Profile of Moods State (POMS); Sick, Control, One stone, Fat, Food (SCOFF); Sleep quality and quantity; Perception of Success Questionnaire (PSQ); Passion for Dance Scale; Additional questionnaire: amount of dance and other physical activity engaged in over the previous month, details of warm up, cool down, and environmental factors (flooring, footwear, physical dance environment). Baseline physical screening protocol: anthropometry, lower limb flexibility and power, foot	dance). Correlations between (a) the proportion of weeks in which sleep quality was rated very good/good and the number of days missed/impeded during injury, and (b) the proportion of weeks in which general health was rated very good/good and the number of days missed/impeded during injury.	injury, onset was sudden or gradual. Subjective outcomes questionnaire: data on physical (amount of dance and other physical activities engaged in over the previous month, details of warm up and cool down), environmental (satisfaction with the dance environment - temperature, noise, lighting, etc), psychological (mood - Profile of Moods State; pain beliefs - CSQ; achievement motivation - PSQ; passion for dance - Passion for dance Scale), and general health and wellbeing (SHCI) factors, in addition to injury status; also disorder eating (SCOFF) and sleep	or completely unable to dance that week).

	McGuinness et al. (2006)	Noon et al. (2010)	Stein et al. (2013)	Cahalan et al. (2013)	Eustergerling et al. (2015)	Cahalan et al. (2015)	Cahalan et al. (2016)	Cahalan, Bargary et al. (2018)	Cahalan, Kearney et al. (2018)	Cahalan, Comber et al. (2019)	Cahalan, Bargary et al. (2019)
								alignment, single leg balance, hypermobility, cardiovascular fitness and endurance, leg dominance and functional movement tests.		quality and quantity. Physical Screening: measures of anthropometry , lower limb flexibility, and power, foot alignment, balance, hypermobility, cardiovascular fitness and endurance, leg dominance, and a nr of functional movement tests.	
**Inclusion/exclusion criteria**	A convenience sample of all subjects who could be approached by the researcher during the competition (North American Irish Dance Championships , July 2-7, 2004) were invited to complete a questionnaire	female IDs in a single ID company. Injuries sustained during activities other than dance were excluded	Irish dance injuries that were either directly related or partially related were included	Current and retired professional dancers; aged 18 yrs or +; have spent at least 8 months of a consecutive 12-month period working exclusively as a paid Irish dancer; good understanding and command of written English	Dancers were included in the study if they were registered in an accredited dance school in Calgary; were 12 years or older as of January 1, 2007; and either actively competing, unable to compete due to an injury, or on a 6-month probation for changing dance schools.	not reported	Professional dancers were required to have spent at least 8 months of a consecutive 12-month period working as a paid ID. Competitive dancers must have competed in solo competition within the previous 5 years at the World Championships or relevant national Championships . Student dancers were those in full-	Subjects were required to be actively competing at open (elite) level for a period of at least 1 year	Inclusion criteria required participants to be full-time students of either CD or ID, to be 18 years of age or older with a good command of spoken and written English.	Inclusion criteria required participants to be full-time students of either CD or ID, 18 years of age or older, and have a good command of spoken and written English. Participants were also required to be injury- free in order to be able to complete the baseline physical testing element of the assessment.	Participants were required to be actively competing at open (elite) level for a period of at least 12 months

	McGuinness et al. (2006)	Noon et al. (2010)	Stein et al. (2013)	Cahalan et al. (2013)	Eustergerling et al. (2015)	Cahalan et al. (2015)	Cahalan et al. (2016)	Cahalan, Bargary et al. (2018)	Cahalan, Kearney et al. (2018)	Cahalan, Comber et al. (2019)	Cahalan, Bargary et al. (2019)
							time university education studying ID				
**Number of injuries**	191 reported injuries	217, nº of total injuries per dancer 1 to 17, with a mean of 3.1 injuries per dancer (a repeat injury was counted as a second injury)	437, Nº of diagnoses, mean (range) 1.7 (1 – 7) diagnosis/dancer	396	60	153	278	63	88; 4.2(2.5) injuries per dancer (SD) ID group	58 (ID group)	130
**Injury estimates (incidence, prevalence, time exposure)**	125 (79%) of the dancers suffered 1 or + injuries; 75 (60%) experienced 1 injury; 34 (27%) 2 injuries; 16 (13%) 3 injuries; 34 (21%) had no injuries	The nº of total injuries per dancer ranged from 1 to 17, with a mean of 3.1 injuries per dancer. Nº of total injuries/ level of dancer were 26, 66, and 125 injuries for levels 3, 4, and 5, respectively.	not applicable	137 dancers (76.9%) reported a previous injury, with 41 (23.1%) reporting no injury; mean career injury rate for all dancers 2.25±2.04	26 (72.2%) dancers reported at least 1 Irish dance-related injury in the previous 12 months.	Proportion (5y) = 80 (76.9%) under time-loss definition. Mean of injuries sustained to all body parts over the previous 5y = 1.49±1.2; 337 complaints	82.1% reported some level of pain/injury throughout the year of the study	84% of subjects recorded at least one pain or injury during the previous year. Of the 37 subjects, 16.2% reported no incidence of pain and injury during the preceding year; 18.9% suffered one episode; 48.7% reported 2 episodes; 10.8% reported 4 episodes; 5.4% reported more than 4 episodes.	When injuries are defined as self-reported injury: incidence of 10.6 injuries/1000 hours dancing. The time-loss incidence= 3.4 injuries/1000 hours dancing. ID group	At least one episode of injury was reported by 92.6% of IDs	prevalence=86, 5%, incidence for all injuries was 9.3 injuries/1000 h danced, incidence (time-loss injuries) = 4.5 injuries/1000 h danced
**Nature of injuries/Aetiolo gy**	The majority of the injuries occurred before a major competition (n = 111; 58%) or when new steps were introduced (n = 41; 21%).	The majority of injuries were overuse	20.4% acute or traumatic injuries, 79.6% overuse	64 (49.2%) PIDs had been injured mid-way through a production run, while 27 (20.8%) and 26 (20.0%), respectively, sustaining their	not reported	Self-perceived causes of pain and injury: overuse (30%), accident (14.6%), and inadequate warm-up and stretching (12.3%), muscle weakness (10.8%), generalized fatigue	The main perceived causes of pain/injury reported by subjects themselves were overuse and fatigue (32.5%),	not reported	Perceived causes of injuries: unclear/unkn own - 57,5%, Overuse 19,5% accident 9,2%	Sudden onset: 11 (33.3%); Gradual onset: 12 (36.4%); Unsure/not stated: 10 (30.3%)	Participants perceived that overuse/ excessive dancing accounted for 42.9% of all episodes of injury, with accident

	McGuinness et al. (2006)	Noon et al. (2010)	Stein et al. (2013)	Cahalan et al. (2013)	Eustergerling et al. (2015)	Cahalan et al. (2015)	Cahalan et al. (2016)	Cahalan, Bargary et al. (2018)	Cahalan, Kearney et al. (2018)	Cahalan, Comber et al. (2019)	Cahalan, Bargary et al. (2019)
				most serious injury near the end of tour and in the early stages of touring. Only 14 PIDs (10.8%) stated that they had become injured in the rehearsal period of the production run. Of the six career-ending injuries, one occurred during the rehearsal period, two in the early stages of the tour, and three near termination of the production run.		(6.2%), and lapses in concentration (2.3%), unsuitable floor surfaces (7.7%), footwear (3.8%), and difficulties with choreography (2.3%)	accidents (15.6%), previous injury (13.2%), poor stretch/warm-up (11.3%) and a number of biomechanical factors such as muscle weakness and growth spurts (11.3%). Other factors cited less often included unsuitable floors, older age, difficult choreography, stress, and cold ambient conditions				(11.5%), growth (8.4%) and choreography technique (8.1%) also identified as common factors.
**Anatomic injury sites**	Ankle (n=60; 32%); foot (n=48;25%); hip (n=23;12%); knee (n=19; 10%); upper limb (n=4;2%), back injuries (n=10;5%); other (n=27; 14%)	Lower extremities (94.9%), lumbosacral spine and pelvis (5.1%); most injuries were in the foot and ankle 68.3%	Foot 33%, Ankle 23%, Knee 20%, Hip 14%, Leg 5%, Back 4%, Other 20%	head/face, 1,77%; neck, 6,31%; arms/hands, 2,02%; shoulders, 2,53%; upper back, 1,77%; lower back, 7,32%; pelvis, 1,52%; ribs, 2,02%; hip, 4,55%; groin, 4,04%; thigh, 3,79%; knee, 9,6%; calf, 8,33%; ankle, 21%; foot, 23,49%. Lower limb accounted	Foot=36.7%; ankle =30.0%; lower leg=6.7%; knee=10.0%; upper leg=1.7%; hip=13.3%; wrist=1,7%	Foot/ankle=48.8%, calf/shin=11.3%, knee=10.8%, hip/thigh=9.4%, lower back=6.4%, head/neck=4.3%, elbow/wrist/hand=2.5%, groin=2.0%, ribs=2.0%, coccyx=1.0%, pelvis=0.5%, abdomen=0.5%, upper back=0.5%	foot and ankle 53 (63.1%), knee 20 (23.8%), shin 12 (14.3%), calf 10 (11.9%), thigh 9 (10.7%), lower back 5 (6.0%), hip 8 (9.5%), groin 1 (1.2%)	Foot and ankle=42.8%, knee=11.1%, calf=9.6%, hip=7.9%, lower back=7.9%, thigh=6.3%, shin=4.8%, groin=4.8%, buttocks=3.2%, shoulder=1.6%	lower back 9,1%, shoulder 2.3%, neck 6.8%, upper back 4.5%, head 3.4%, mid back 1.1%, chest 1.1%, ribs 1.1%; knee 15,9%, thigh 11,4%, groin 3.4%, shin 4.5%, buttocks 6.8%, calf 3.4%, foot/ankle	lower back, 5.0%; head and neck, 3.3%; mid-back, 3.3%; shoulder, 3.3%; wrist and hand, 1.7%); knee, 10.0%; shin, 8.4%; thigh, 10.0%; calf, 10.0%; groin, 3.3%; hip, 5.0%; buttocks, 1.7%; foot and ankle, 30.0%	lower back, 11.4%; ribs, 0.8%; shoulders, 0.8%; upper back, 0.8%; thigh, 12.3%; groin, 10.8%; knee, 10.8%; calf, 7.7%; shin, 5.4%; buttocks, 4.6%; foot/ankle, 34.6%

	McGuinness et al. (2006)	Noon et al. (2010)	Stein et al. (2013)	Cahalan et al. (2013)	Eustergerling et al. (2015)	Cahalan et al. (2015)	Cahalan et al. (2016)	Cahalan, Bargary et al. (2018)	Cahalan, Kearney et al. (2018)	Cahalan, Comber et al. (2019)	Cahalan, Bargary et al. (2019)
				for 296 (74.7%) of all injuries: 93 (67.9%) dancers had a foot injury and 83 (60.6%) dancers had an ankle injury					23,9%, toes 1.1%		
**Types of injuries/symptoms**	Ankle sprains (n=56; 29%); ankle fracture (n=4; 3%); foot stress fractures (n=22; 12%); foot sprain (n=15; 7%); plantar fasciitis (n=4; 2%); hip sprain (n=9; 5%); hip bursitis (n=8; 4%); groin strain (n=3; 1.5%); ITB tightness (n=1; 0.5%); knee sprain (n=11; 6%); PFP (n=4; 2%); meniscal tear (n=2; 1%); knee tendonitis (n=2; 1%); wrist fracture (n=2; 1%); wrist sprain (n=2; 1%); facet joint strain (n=9; 4,5%); prolapsed disc (n=1; 0.5%)	Stress fractures comprised nearly a third (29.9%) of all injuries, patellofemoral pain syndrome (PFPS) (11.0%), Sever condition (6.0%), ankle sprains (5.1%), posterior tibialis tendonitis (4.6%), and plantar fasciitis (4.6%) were the most common injuries. The most common sites for stress fractures were the sesamoids (27.7%) of total stress fractures), metatarsals (23.1%),	66.4% of all diagnoses fit into one of these seven categories: tendon injury (13.3%), apophysitis (11.4%), patella pain or instability (10.8%), stress injury (10.1%), muscle injury (7.8%), ligament injury (7.3%), or fracture (excluding stress fractures: 5.7%). The category general pain, weakness, or swelling (which accounted for 8.9% of all diagnoses) was not used in analysis. Stress injuries (including medial tibial stress syndrome, stress reaction, and stress fracture) accounted for 10.1% of the total diagnoses. These injuries involved the foot (52.3%), leg (25.0%), ankle (15.9%), and back (6.8%)	Structures injured were split evenly between muscle (27%), bone (25%), ligament (24%), and tendon (22%), with cartilage and nerve injuries accounting for less than 2% of the self-reported injured structures cumulatively	not reported	Unaware of diagnosis=19.2%, muscle strain=17.2%, joint sprain=12.6%, fracture=10.2%, ligament=9.6%, tendonitis=6.0%, stress fracture=6.0%, joint trauma=6.0%, unspecific bony injury=1.%, cartilage=1.2%, other=10.6%	not reported	Muscular damage or weakness 18 (36.0%), Ligament damage 10 (20.0%), Tendinopathy 4 (8.0%), Shin splints 4 (8.0%), Plantar fasciitis 3 (6.0%), Bony oedema 3 (6.0%), Biomechanical factors (posture and foot alignment) 3 (6.0%), Stress fracture 2 (4.0%), Other (fracture, osteochondrom, os trigonum) 3 (6.0%)	Muscle n=16 (18.4%); Tendinopathy n=1 (1.1%) 9,1%; Joint n=5 (5.7%); Sprain/Ligament n=3 (3.4%); Meniscus, Fracture, Fasciitis, Bursitis n=1 (1.1%); Nerve n=3 (3.4%); Impingement n=3 (3.4%); Infection n=3 (3.4%); WAD/Concussion n=1 (1.1%); Unclear/unknown n= 50 (57.5%); Shin splints n=1 (1.1%)	Muscular strain or fatigue accounted for 50% of all self-diagnosed injuries reported, and 26.0% of injuries were diagnosed by medical professionals. Ligament damage, tendon issues, and metatarsalgia were also commonly reported by participants who either self-diagnosed their injury or were diagnosed professionally. Most injuries reported by both groups were recurrences (58,6%) of a previous complaint. Irish dancers: Recurring=58.6 %, New=41.4%	Twelve injuries (9.2%) had multiple diagnoses recorded and three injuries had no diagnosis recorded. Of the remaining 115 injuries, the majority (58.3%) had an unclear/unknown diagnosis, while a further 20.9% were muscular in nature. Joint pathology (7.8%), tendinopathy (6.1%), shin splints (2.6%), fracture (1.7%), inflammation (1.7%) and infection (0.9%)

	McGuinness et al. (2006)	Noon et al. (2010)	Stein et al. (2013)	Cahalan et al. (2013)	Eustergerling et al. (2015)	Cahalan et al. (2015)	Cahalan et al. (2016)	Cahalan, Bargary et al. (2018)	Cahalan, Kearney et al. (2018)	Cahalan, Comber et al. (2019)	Cahalan, Bargary et al. (2019)
		navicular (12.3%), first proximal phalanx (12.3%), and tibia (9.2%). Of the metatarsal stress fractures, the majority was seen in the first metatarsal, representing 9.2% of total stress fractures and 40% of total metatarsal stress fractures. The next leading metatarsal stress fracture was at the second metatarsal, representing 6.2% of total stress fractures and 26.7% of total metatarsal stress fractures. Nine of the 69 dancers had recurrence of the same injury.									
**Injuries severity**	*65% (n =124) of injuries took 21 days or	not reported	not reported	223 (56.3%) minor (1-7days), 90	not reported	Time-loss: minor up to 7 days to resolve; moderate:	not reported	22.2% were minor (requiring up to 7 days to resolve); 39.7% were moderate (between 8-21 days);		days lost) 1-7 days (mild): 28 (48.2%); 8-21	90 (69.2%) injuries were mild in nature

	McGuinness et al. (2006)	Noon et al. (2010)	Stein et al. (2013)	Cahalan et al. (2013)	Eustergerling et al. (2015)	Cahalan et al. (2015)	Cahalan et al. (2016)	Cahalan, Bargary et al. (2018)	Cahalan, Kearney et al. (2018)	Cahalan, Comber et al. (2019)	Cahalan, Bargary et al. (2019)
	more to recover and resume dancing			(22.7%) moderate (8-21 days), 77 (19.4%) severe, 6 (0.2%) were career ending; 3 (1.7%) participants reported that they never danced in pain, while 35 (19.7%) rarely did; 8 dancers reported pain while dancing sometimes (44.9%) and 60 (33.7%) often or always danced in pain.		between 8-21 days; severe: longer than 21 days to resolution; 24 (15.7%) were minor, 42 (27.4%) were moderate, and the remaining 87 (56.9%) were severe		38.1% were severe (took longer than 21 days to resolve). Of the 63 episodes: 57.1% cases subjects were completely unable to participate in dance for at least the full day following the event; in 25.4% episodes of pain and injury subjects were able to participate in at least some aspects of dance training; in 11.7% episodes, subjects were able to participate fully in dance; 50.6±84.0 days lost to pain and injury		days (moderate): 15 (25.9%); 21+ days (severe): 15 (25.9%). Dance status when injured: Not able to dance: 14 (24.1%); Partially able to dance: 26 (44.8%); Able to dance fully: 18 (31.1%)	and resolved within one week, 24 (18.5%) injuries were moderate and had resolved within four weeks, 16 (12.3%) were severe, requiring longer than four weeks to resolve. The median duration for an injury was 1 (IQR= 1, 2) week.
**Most common injuries**	Ankle sprains (n=56; 29%); foot stress fractures (n=22; 12%)	The top injuries included stress fractures (29.9%), patellofemoral pain syndrome (11.1%), Sever condition (6.0%), ankle sprains (5.1%), posterior tibialis tendonitis (4.6%), and plantar fasciitis (4.6%)	Tendon injury (13.3%), apophysitis (11.4%), PFP or instability (10.8%), stress injuries (10.1%), muscle injury (7.8%)	Muscle (27%), bone (25%), ligament (24%), and tendon (22%)	foot and ankle	Unaware of diagnosis=19.2%, muscle strain=17.2%, joint sprain=12.6%, fracture=10.2%. Foot and ankle injuries were most common	foot and ankle	Foot and ankle. Muscular damage or weakness=36.0 %; ligament damage=20.0%	Foot and ankle; Muscle Injuries (18,4%); joint/sprain ligament (9,1%)	Foot and ankle. Muscular strain or fatigue accounted for 50% of all self-diagnosed injuries reported, and 26.0% of injuries	foot/ankle (n=45, 34.6%)
**Associated risk factors**	There were no differences	As the IDs increased in	traumatic injuries were more likely to involve	Those who had missed a	Dancers at an elite level were	Female sex, a high number of	Factors significantly	statistically significant	Better sleep (p=0.007) and	not reported	An increased risk of injury

	McGuinness et al. (2006)	Noon et al. (2010)	Stein et al. (2013)	Cahalan et al. (2013)	Eustergerling et al. (2015)	Cahalan et al. (2015)	Cahalan et al. (2016)	Cahalan, Bargary et al. (2018)	Cahalan, Kearney et al. (2018)	Cahalan, Comber et al. (2019)	Cahalan, Bargary et al. (2019)
	between dancers with and without ankle injuries in demographics (e.g., age, years, hours per week of practice, hours per week of classes, or competitions per year). Most of the injuries occurred before a major competition (n = 111; 58%) or when new steps were introduced (n = 41; 21%). Significant difference between dancers with and without ankle injuries in relation to the use of split sole sneakers, warm up, cool down (p<0.0001).	level of competition and number of hours practiced per week, so did the number of injuries per dancer.	the right side; An association was found between age at diagnosis and the seven diagnostic categories (p = 0.014); dancers diagnosed with apophysitis were on average 3 years younger than those diagnosed with stress injury (p = 0.007); injury type (trauma or overuse) was associated with time to presentation (p < 0.001), with traumatic injuries presenting sooner than those due to overuse	performance due to injury were significantly older (p = 0.008) and more experienced (p = 0.002) than those who had not. There was a weak correlation between the nº of yrs of experience as a PID and the total nº of injuries incurred (rs =0.251, p = 0.001).	more likely to report at least one injury than dancers who were non-elite (OR=6.33, CI 1.27–31.57). In addition, dancers who were over age 18 were more likely to report an injury than dancers who were under 18 yrs of age (OR=24.3, CI 2.60–229.56). No significant differences were found in risk of injury for years of participation in Irish dance, participation in physical activities or sports other than Irish dancing, or performing a warm-up or cool-down	subjective general and psychological health complaints, low mood, higher catastrophizing, and a reported failure always to warm-up were significantly associated with musculoskeletal pain and injury	associated with membership of the MTA group included greater anger-hostility (P = 0.003), more subjective health complaints (P = 0.026), more severe previous pain/injury (P = 0.017), more general everyday pain (P = 0.020), more body parts affected by pain/injury (P = 0.028), always/ often dancing in pain (P = 0.028), and insufficient sleep (P = 0.043).	greater number of troublesome body parts (p= 0.002), higher levels of anger and hostility (p = 0.045), and a higher level of often or always dancing in pain (p = 0.033)	general health (p<0.001) scores were negatively correlated with days lost/impacted by injury		was associated with poorer general health and reduced dance exposure, but not with sleep quality or change in weight or height.
**Main limitations & recommendations**	Limitations: the data for this study were collected on the 3 most recent injuries in the previous 5 years and this did not allow for the calculation of a yearly injury	not reported	Limitations: data extracted from medical records, with incomplete information regarding dancers' level of dance or frequency/duration/intensity; few charts had information on whether other providers, including athletic trainers, massage	Limitations: errors and omissions due to subject recall; absence of definition of "accident" and any associations with fatigue or overuse injuries or	Limitations: nature of the self-report survey; under-reporting of injury related to recall bias; influence of a significance of an event, some injuries are more readily	Limitations: study required subjects to recall events over a 5-year period, errors of omission and recall are inevitable, and the frequency of minor injuries is most likely understated. Impossible to calculate an	Limitations: small sample size, days absent from dancing because of illness were not factors into the analysis, examining the role of physical factors specific	Limitations: small sample size, as a retrospective study, errors of recall and omission are possible, and it is likely that some minor injuries were omitted.	(1) sample size is low (n=21); (2) data are largely self-reported, thereby open to interpretation and error; (3) The unique characteristics	Limitations: small sample, recall bias, several questionnaires used in this study have not been previously validated in a dance population.	Limitations: small sample; tools to collect general health and sleep data were relatively crude; only an external measure of dance exposure (hours of

	McGuinness et al. (2006)	Noon et al. (2010)	Stein et al. (2013)	Cahalan et al. (2013)	Eustergerling et al. (2015)	Cahalan et al. (2015)	Cahalan et al. (2016)	Cahalan, Bargary et al. (2018)	Cahalan, Kearney et al. (2018)	Cahalan, Comber et al. (2019)	Cahalan, Bargary et al. (2019)
	rate; minor injuries (less than 2 weeks injured) not reported; not questioned about other types of dance. Recommendations: a more detailed investigation into the exact type of surface the subjects in this population used is needed.		therapists, physical therapists, chiropractors, or other physicians had been consulted before the appointment at the investigators' hospital	trauma were not established; other potential causes of injury not fully addressed (age at time of injury, sleep quality, the role of footwear, physical characteristics, and the female athlete triad. Recommendations: focus on prospective studies of Irish professional dancers, involving physical and psychosocial elements, with a view to generating an appropriate screening process and general model to predict those at risk of injury.	recalled than others; target sample size; possible type II error; non-competitive dancers or those within other regions may have different characteristics; low nr of male dancers - not feasible to make comparisons between male and female dancers. Recommendations: screening programs should consider demographic, lifestyle, and psychosocial factors, as well as the physical traits that affect performance	accurate response rate, which may be indicative of volunteer bias and a study cohort that is potentially non-representative of the wider elite ID population. Some elements of the functional screening were basic and generic and would benefit from greater specificity to IDs. Anonymity of the questionnaire data could have allowed for more candid responses, particularly regarding drug and alcohol use and food consumption. Greater detail of the type and duration of warm up and cool down routines would have been beneficial. Recommendations: a prospective study was undertaken by the investigators.	to each bodily area may show greater predictive ability, did not identify those subjects who may have pain but continued to dance regardless. Recommendations: precise, discrete definitions of pain and injury would be beneficial; screening protocols should be developed to identify IDs at risk for pain/injury. Clinicians should record and monitor incidences of illness as well as pain/injury under clearly defined parameters.	Recommendations: education for teachers, dancers, and parents concerning the risk factors for pain and injury in young IDs is advocated.	of subjects may not be adequately explored by the factors investigated. 4) tools used for sleep and general health are relatively crude; 5) and data regarding previous cross-training activity were not collected; 7) The use of a four-week gap to categorise an event as a new injury was arbitrarily decided based on the clinical experience of the investigating team; 8) more expansive definition of training load combining an internal measure of exercise intensity such as rate of perceived exertion, and an external measure of activity such as hours dancing, would be preferable to	Recommendations: Indications of a shared dancer mentality including ego-driven motivation, passion for dance, and lower mood were present and required vigilance on the part of teachers and coaches to mitigate possible negative physical and psychological consequences	dance) was collected; severity of injury was applied both to days when the participants were unable to dance or only partially dance; the choice of a four-week gap to identify an event as a new injury was arbitrarily chosen based on the clinical experience of the researchers, and may be inaccurate in some cases. Recommendations: Education for clinicians and dance practitioners on load monitoring, periodisation and tapering is recommended, with avoidance of sudden peaks in training load to ameliorate injury risk. Appropriate cross-training, which was underutilised by this cohort, may provide an

	McGuinness et al. (2006)	Noon et al. (2010)	Stein et al. (2013)	Cahalan et al. (2013)	Eustergerling et al. (2015)	Cahalan et al. (2015)	Cahalan et al. (2016)	Cahalan, Bargary et al. (2018)	Cahalan, Kearney et al. (2018)	Cahalan, Comber et al. (2019)	Cahalan, Bargary et al. (2019)
									hours of dance alone		opportunity for enhanced physical fitness and reduction of injury risk.
**Comments**	* different values in abstract	50% of the dancers were 15 years of age and had not yet reached menarche. 40% of the dancers were eumenorrheic defined as regular cycling every 28-30 days. Four of the 67 dancers had recently reached menarche and menstrual status had not been established. 1 dancer was oligomenorrheic, defined as cycles 38 days apart 2 years after menarche.1 dancer reported 17 menstrual cycles in the last 12 months. Introduction does not have references	The most common treatment was physical therapy (84.3%); majority of dancers (58%) had a single diagnosis; dancers' injury prevention suggestion: stretching 69.8%, strengthening or conditioning 24.8%, use of shoe support 14.7%, educating dance teachers 12.4%, cross training 10.1%, educating dancers 8.5%, use of sprung floors 8.5%, age appropriate dance and rest 4.7%, nothing can be done to prevent injuries 3.1%, don't know 3.1%	among the 169 dancers who responded to questions regarding psychological problems throughout their careers: 24% had no psychological problems, 20% reported either one or two; main problems were tension with people (47.3%), stress due to external factors (30.8%), general anxiety (29%), performance anxiety (26.6%); The most popular preventive measure taken was to seek professional help (92 dancers, 67.1%); most dancers danced in pain - difficult to access overuse injuries.	This paper misses tables and/or chart to present the data	most subjects in this study had healthy BMI values and waist:hip ratios. Physical traits were not found to be significantly associated with injury, but physical variables were normal	This cohort was part of a larger subject group of 104 dancers, 19 of whom did not wish to participate in the 12-month follow-up period. Could have monitored dance exposure to calculate incidence	There was no association between any physical trait assessed and pain and injury status. Proposed explanations include inappropriate technique progression, unique choreographic features, and an overly arduous calendar of competitive events. though as a retrospective study it is impossible to establish causation.	IDs engaged in an erratic calendar of dance exposure, with sudden spikes in hours danced.		Lower weekly hours of dance practice than other genres with little evidence of periodisation are evident in this cohort. However, increased dance load appears to be protective against injury in these participants. 63 of the 130 injuries (48.5%) were time-loss injuries, indicating that dancers still danced either partially or fully while injured in over 50% of cases. In the current study, there was no association found between sleep quality and injury BUT participants largely enjoying good/very good quality sleep.
